# *Staphylococcus aureus* colonization and bloodstream infection in very preterm infants

**DOI:** 10.1080/19490976.2025.2592423

**Published:** 2025-12-02

**Authors:** Rebecca L. Knoll, Daniel Podlesny, Ingmar Fortmann, Wolfgang Göpel, Michael Zemlin, Susan Lynch, Peer Bork, Stephan Gehring, Christoph Härtel

**Affiliations:** a Department of Pediatrics, University Hospital Mainz, Mainz, Germany; b Benioff Center for Microbiome Medicine, Department of Medicine, University of California San Francisco, San Francisco, CA, USA; c Molecular Systems Biology Unit, European Molecular Biology Laboratory, Heidelberg, Germany; d Department of Pediatrics, University Hospital Schleswig-Holstein Campus Lübeck, Lübeck, Germany; e Department of General Pediatrics and Neonatology, Children's Hospital, Saarland University, Homburg, Germany; f Department of Pediatrics, University Hospital Würzburg, Würzburg, Germany

**Keywords:** Staphylococcus aureus, colonization, bloodstream infection, metagenome

## Abstract

**Background:**

Staphylococcus (S.) aureus remains a frequent pathogen for neonatal late-onset bloodstream infections (BSIs). The impact of colonization screening on BSI incidence is less understood.

**Methods:**

We assessed the epidemiology of late-onset S. aureus BSI in two independent multicenter cohorts of preterm infants born at < 33 weeks' gestation, the German Neonatal Network (GNN, very low birth weight infants) and PRIMAL (infants with a gestational age 28−32 weeks). In the PRIMAL cohort, we determined S. aureus colonization in fecal samples by culture and shotgun metagenomic sequencing (metaG) during the first year of life. In addition, we integrated publicly available metaG data from preterm infant cohorts born at 23–34 weeks' gestation.

**Results:**

Late-onset S. aureus BSI was noted in 1.5% (336/21491) in preterm infants in the GNN cohort and 0.5% (3/638) in the PRIMAL cohort, respectively. At day 30 of life, 7.6% (42/553) of fecal samples were positive for S. aureus, while available metaG data of corresponding samples revealed S. aureus positivity in 36.6% (159/434). Every 10-fold increase in S. aureus relative abundance (metaG) was associated with a 2.9-fold higher odds of S. aureus detection in blood culture. We also confirmed *S. aureus* detection in 22% (393/1782) of samples across several published cohorts of preterm infants by metaG, while 95 samples carried at least one *Staphylococcus*-specific virulence gene (SVG).

**Conclusion:**

Our study demonstrates that metagenomic quantification of pathobionts such as *S. aureus* in intestinal samples provides a stronger predictor of colonization than culture. Future prevention strategies should focus on promoting S. aureus colonization resistance through microbiome-informed approaches.

## Introduction

U.S. network data recently confirmed that *Staphylococcus (S.) aureus* is still a problematic pathogen for neonatal late-onset bloodstream infections (BSIs) affecting 2% of very low birth weight infants (VLBWI; birth weight <1500g) and contributing to 14% mortality.^1^ Despite decades of awareness, progress in reducing infection rates remains limited and calls for renewed collaborative efforts. Here, we contribute epidemiological data from the German Neonatal Network (GNN), along with colonisation surveillance and high-resolution microbiome profiling from the PRIMAL cohort,^2^ supplemented by publicly available microbiome data from U.S. cohorts.^3-7^

## Methods

We assessed late-onset *S. aureus* BSI in two independent multicenter cohorts of preterm infants born at <33 weeks' gestation (a) the GNN, an ongoing population-based study of VLBWI, and (b) the PRIMAL study which included surveillance culture results and deep metagenomic sequencing (metaG) of faecal samples collected at three scheduled timepoints: day of life (DOL) 1, 30, and 356. To expand the dataset, we integrated publicly available metaG data from the SPIRE platform,^8^ including data from preterm infants born at 23–34 weeks' gestation.^3-7^ All sequencing data were processed using a unified bioinformatic pipeline.^8^ Read assemblies were mapped against the virulence factor data base (VFDB) for known *Staphylococcus* virulence genes.^9^ To account for stochastic detection effects due to varying sequencing depths, prevalence of *S. aureus* and virulence genes was normalised to standardised sequencing depths (minimum, maximum, 1 st quartile, median, and 3rd quartile) derived from the distribution of sequencing depths across all metagenomic samples (Supplementary Figure 1).

## Results

In the GNN cohort, the incidence of *S. aureus* BSI was 1.5% (Table 1) and remained unchanged after routine *S. aureus* colonisation screening was officially recommended in 2013, i.e. before screening era, 2009−2012: 90/5377, 1.6%; after official recommendation 2013−2023: 238/16089, 1.4%; *p* = 0.4). During these time frames we also noted an increased use of central lines (58.4 vs. 72.8% VLBWI, *p* < 0.001), with central line use being more common among infants with BSI (77.1%) than in the overall cohort (69.1%; *p* < 0.001, Fisher’s exact test). In parallel, we observed faster advancements to full enteral feeding, i.e. enteral tolerance of 150 ml/kg body weight per day (median/IQR, 2009−2012: 13/10 vs. 2013−2023: 12/9 days, *p* < 0.001, Mann-Whitney-U test).

**Table 1.** Clinical characteristics of GNN and PRIMAL cohort.

Variable	GNN, with *S. aureus* BSI (*n* = 336)	GNN, all (*n* = 21491)	PRIMAL, all (*n* = 638)
Gestational age, weeks	26.0 (24.6−28.1)	27.7 (25.9−29.6)	31.0 (29.7−32.1)
Birth weight, g	831 (640−985)	960 (730−1220)	1475 (1240−1760)
Sex, female	146 (43.5)	10341 (48.1)	294 (46)
male	190 (56.5)	11144 (51.9)	344 (54)
*S. aureus* LOS BSI	336 (100)	336 (1.5)#	3 (0.5)
<500g	31 (9.2)*	31/1248 (2.5)#	
500-749g	134 (39.9)*	134/4779 (2.8)#	
750-999g	104 (30.9)*	104/6386 (1.6)#	
1000-1249g	40 (11.9)*	40/4571 (0.9)#	
1250−1499 g	17 (5.1)*	17/4498 (0.4)#	
MRSA BSI	24 (7.1)	24 (0.1)	1 (0.2)
Central line	259 (77.1)	14078 (69.1)	338 (53)
Advancement to full feeds (150ml/kg/d), days	15 (11−24)	13 (9−18)	10 (7−15)
Vancomycin	239 (71.1)	7213 (33.6)	23 (3.6)
Teicoplanin	39 (11.6)	1470 (7.6)	40 (6.3)
All-cause Mortality	16 (4.8)	880 (4.1)	1 (0.2)
BSI related death	7 (43.8)§	167 (20.1)§	
Death after day 7	15 (93.8)§	619 (70.3)§	

GNN is a population-based cohort of very low birth weight infants (VLBWI; 68 participating sites; database 2009–2023; *n* = 21491). During the observational study, routine colonisation screening was officially recommended and implemented at all sites in the year 2013. The PRIMAL randomised controlled trial evaluated the effects of probiotic supplementation (*Bifidobacterium longum* subsp. *infantis*, *B. animalis* subsp. *lactis* BB−12, *and Lactobacillus acidophilus* La−5) on microbiome development in infants born at 28–32 weeks' gestation (18 sites; 2018–2020; *n* = 638). Both studies received ethical approval from institutional review boards at participating sites. Variables are described in median (IQR) for continuous measures or *n* (%) for binary parameters. * proportion of affected infants per birth weight group, # incidence of late-onset *S. aureus* BSI in birth weight group, § proportion within the group of non-survivors.

**Figure 1.**
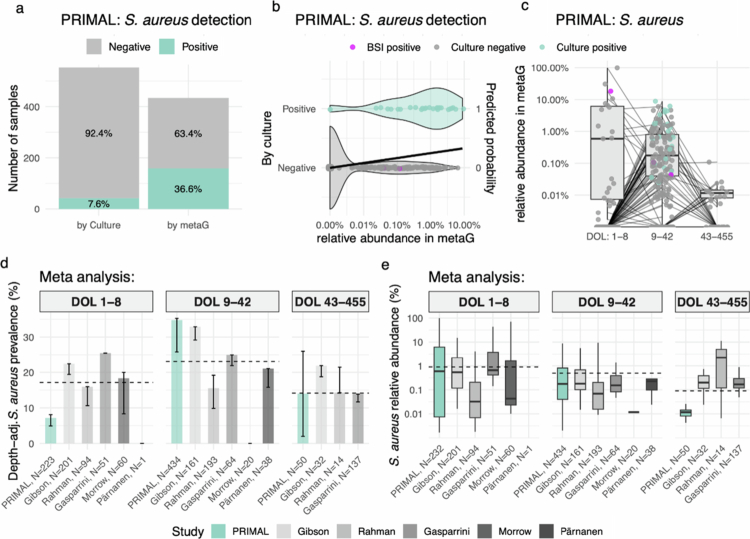
Detection and dynamics of *Staphylococcus aureus* colonisation across early life and multiple cohorts. **a**, Total number of stool samples tested for *S. aureus* using culture (*N* = 553) and metaG (*N* = 434) in the PRIMAL cohort at DOL 30. Bars are colour-coded by detection status (positive or negative), with the proportion of positive samples displayed as percentages within each bar. **b,** Logistic regression of *S. aureus* culture detection at day of life 30 in the PRIMAL cohort. The x-axis shows log*10* -transformed relative abundance in metagenomic stool samples (pseudocount *10^*−5 was added before log-transformation). The right y-axis depicts the predicted probability of culture positivity (black regression line), while the left y-axis shows culture result (negative vs. positive) with violins indicating the distribution of abundances by detection status. Points represent individual samples. A 10-fold increase in relative abundance was associated with ~3-fold higher odds of culture positivity (OR = 2.92, *p* < 0.001). **c,** Relative abundance of *S. aureus* in stool samples from the PRIMAL cohort and stratified by postnatal age intervals (Day of life (DOL) 1–8, 9–42, 43–455). Samples from infants who developed late-onset *S. aureus* bloodstream infection (BSI) are highlighted in magenta, for DOL. Samples that were culture positive around DOL 30 are coloured in teal. Lines connect samples from the same study subject. **d**, Prevalence of *S. aureus* colonisation (mOTU count > 0) across cohorts and postnatal age groups. Bars show the percentage of samples with detectable *S. aureus* at median adjusted sequencing depth, with error bars indicating prevalence at the 1 st and 3rd quartiles adjusted sequencing depth. The dashed line marks the overall median prevalence at median adjusted sequencing depth across all cohorts for each age interval. **e,** Relative abundance of *S. aureus* in stool samples across the six independent study cohorts (colour-coded; 3, suppl Table 1) and stratified by postnatal age intervals (DOL 1–8, 9–42, 43–455). Boxplots show the median (line), interquartile range (IQR) (box) and 1.5 × IQR range (whiskers), dashed line represents mean across all studies per age interval.

The incidence of *S. aureus* BSI in the PRIMAL cohort was 3/638 (0.5%), two affected infants were in the probiotic group and one in the placebo group (safety analysis). In line with our previous report,^2^ we did not detect significant differences in *S. aureus* prevalence between probiotic and placebo arms, nor did colonisation with *B. longum subsp. infantis* significantly alter S. *aureus* prevalence or relative abundance (data not shown). Available data from PRIMAL showed *S. aureus* colonisation around DOL 30 in 7.6% (42/553) of preterm infants by culture and in 36.6% (159/434) by metaG (Figure 1a). Most importantly, this analysis revealed for the first time that a 10-fold increase in *S. aureus* relative abundance in the faecal metagenome is associated with a 2.9-fold higher odds of culture positivity (*p* < 0.001; Figure 1b). This association remained robust after adjusting for sequencing depth (Odds Ratio = 2.8). *S. aureus* relative abundance varied substantially across infants, with the highest levels observed early in life (Figure 1c). The only sample available before onset from an infant who developed *S. aureus* BSI showed among highest relative abundance within all samples collected during DOL 1–8 (Figure 1c). We also confirmed *S. aureus* detection in 22% (393/1782) of samples across several published cohorts of preterm infants by metagenomics, highlighting that our findings apply to preterm populations with different ethnical and regional background (Table 2). *S. aureus* prevalence (Figure 1d) and relative abundance (Figure 1e) were broadly consistent across PRIMAL and the publicly available U.S. cohorts (Table 2).

**Figure 2.**
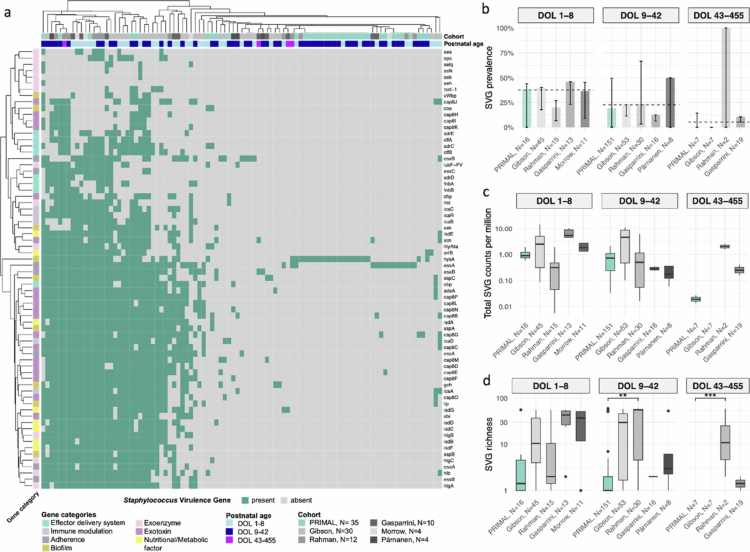
***Staphylococcus***-specific virulence gene (SVG) analysis in metagenomic ***S. aureus***–positive samples* across six study cohorts. a, Heatmap of samples (columns) carrying at least one SVG (*N* = 95) based on median depth–adjusted presence/absence calls. Rows represent individual genes. Colour annotations indicate gene presence (dark teal), postnatal age group, cohort, and virulence gene category. Samples are clustered by virulence gene composition, with shared clusters highlighting co-occurring genes. **b,** SVG prevalence by postnatal age group and cohort, expressed as the proportion of *S. aureus*–positive samples carrying at least one virulence gene. Bars show prevalence at median adjusted sequencing depth; error bars indicate prevalence at the 1 st and 3rd quartiles of sequencing depth. The dashed line marks the overall median prevalence at median depth across all cohorts for each age group. **c,** Total SVG relative abundance per sample, shown as boxplots of summed virulence gene counts normalised to sequencing depth (reads per million). Each box represents the distribution of samples within a cohort and age group (DOL = day of life). Boxplots indicate the median (line), interquartile range (box), and 1.5 × IQR (whiskers). The y-axis is log-scaled. A linear mixed-effects model did not show any significant differences between PRIMAL and the other cohorts. **d,** SVG richness per sample, defined as the number of distinct virulence genes detected based on median depth–adjusted presence/absence calls, stratified by cohort and postnatal age group. Boxplots show the median, interquartile range, and 1.5 × IQR. Significance is shown from linear mixed-effects models (PRIMAL vs. other cohorts), including subject ID as a random factor to account for repeated measures. False discovery rate (FDR) was adjusted using the Benjamini–Hochberg procedure (** = FDR < 0.01, *** = FDR < 0.001). *All analyses were restricted to S. aureus–positive samples, with positivity determined from median depth–adjusted moTU counts.

We next asked whether cohorts differed in *Staphylococcus*-specific virulence gene (SVG) profiles (Figure 2). Among the 393 *S. aureus*–positive samples identified by metagenomics at median sequencing depth, 24.2% (*N* = 95) carried at least one SVG (Figure 2a). PERMANOVA on SVG composition (Jaccard distances) showed that cohort explained 17% of the variance in SVG patterns (*p* = 0.001). When the place of birth (country) was included, it accounted for 12% variance (*p* = 0.001), while a trend was noted for the residual cohort effect (5%, *p* = 0.084). Postnatal age explained little variation and had no significant effect on SVG composition.

In contrast, SVG prevalence declined with increasing postnatal age but was relatively comparable between cohorts (Figure 2b). In addition, total relative abundance of SVGs (Figure 2c) was similar across cohorts. However, the Rahman cohort^4^ showed significantly higher SVG richness than PRIMAL^2^ at DOL 9–42 (linear mixed-effects model with subject ID as random factor, Estimate = 10.4, FDR = 0.00713) (Figure 2d). This suggests that in some cohorts the SVG signal is dominated by a few highly abundant genes, whereas in others a broader repertoire of genes is present at lower abundance, suggesting differences in *S. aureus* strain composition. Notably, all cohorts showed low *S. aureus* and very low SVG prevalence after DOL 43, suggesting a potential hospital-associated effect on *S. aureus* and SVG carriage.

## Discussion

Our study demonstrates that metagenomic quantification of pathobionts such as *S. aureus* in intestinal samples provides a stronger predictor of colonisation than culture. The quantitative link between relative abundance and risk for bloodstream infection (BSI) highlights the potential of metagenomics to improve diagnostics at the level of the individual infant and may also help explain the heterogeneity in *S. aureus* BSI rates observed across NICUs.

Variations in *S. aureus* BSI rates may reflect differences in centre-specific factors e.g. antibiotic use,^10^ enteral feeding practice and infection control bundles,bacterial susceptibility to routine skin disinfectants,e.g. tolerance to chlorhexidine gluconate,^11^^,^^12^ and individual fluctuations in host immunity and microbiome barriers.^13^ Our findings highlight that colonisation with *S. aureus* is consistently found in about 22% of preterm infants in international cohorts, while about one quarter of preterm infants carries known *Staphylococcus* virulence genes. Of note, only a small subset of *S. aureus* colonised infants develop BSI. This suggests that the mere presence of virulence determinants is not the only key driver of invasive disease. Additional host and environmental or strain-specific factors, even only a select subset of virulence genes, likely determine the progression to infection. In line with that, we acknowledge the limitation that we had no information within the publicly available metagenomes who developed BSI caused by *S.aureus*. Furthermore, we cannot confirm a clear relationship between the presence of a central line and the risk of *S.aureus* BSI in the German cohorts. The higher insertion rate of central lines in infants with *S.aureus* BSI may represent a proxy for immaturity-related vulnerability of BSI affected infants (26.0 vs. 27.7 gestational weeks) rather than a causal link. Further large-scale studies are needed to explore the relationship between the prevalence of *S.aureus* at different body sites, e.g. skin and gut, strain specific virulence gene carriage and BSI incidence.

In the PRIMAL cohort, *S. aureus* detection by metaG was more than threefold higher than by culture, indicating that culture-based surveillance may substantially underestimate colonisation. A limitation is that metagenomic sequencing does not provide absolute bacterial quantification in the absence of spike-in controls or cell sorting; thus, our analyses focus on prevalence and standardised relative abundance. Nevertheless, there is accumulating evidence from preterm cohorts^10^^,^^14^ that BSI causing pathogens might be gut derived. Although we could not assess the strain-level origin of BSI pathogens in this study, future work should investigate this important aspect further.

However, given the frequent colonisation but low BSI incidence, broad decolonisation approaches are unlikely to be effective and would require a high number needed to treat, with limited impact on disease rates. Moreover, non-specific decolonisation carries potential risks for VLBWI, including disruption of the developing microbiome that may lead to dysbiosis. Instead, future strategies should focus on promoting colonisation resistance through targeted, microbiome-informed approaches, such as selective probiotic use or phage-based interventions. Further research is needed to understand how the early-life microbiome and host responses modulate the risk of *S. aureus* BSI and to identify the precise window of opportunity, where such interventions could be most effective. 

**Table 2.** Cohort characteristics and sampling overview across six infant faecal metagenomic microbiome studies.

Characteristic	PRIMAL	Gibson	Rahman	Gasparrini	Morrow	Pärnanen
Country of origin	Germany	USA	USA	USA	USA	USA
*N* samples	716	394	301	252	80	39
*N* subjects	466	83	57	38	46	39
*N* samples per subject	2 (1–2)	6 (4.2–10)	5 (5–7)	7 (6–8)	2 (1.8–3)	1 (1–1)
Gestational age in weeks	30.9 (29.4–32.1)	27 (25–29)	30 (28–31)	26.4 (24.8–27)	26 (24.2–28)	32.9 (31–33.4)
C-section	390 (83.7%)	51 (61.4%)	45 (78.9%)	29 (76.3%)	27 (58.7%)	22 (56.4%)
Vaginal	76 (16.3%)	32 (38.6%)	12 (21.1%)	9 (23.7%)	19 (41.3%)	17 (43.6%)
DOL at sampling	28.5 (5–31)	8 (2–22)	13 (7–23)	48 (15.8–203.5)	2.5 (0–8.2)	0 (0–0)
DOL 1−8	232 (32.4%)	201 (51%)	94 (31.2%)	51 (20.2%)	60 (75%)	1 (2.6%)
DOL 9−42	434 (60.6%)	161 (40.9%)	193 (64.1%)	64 (25.4%)	20 (25%)	38 (97.4%)
DOL 43−455	50 (7%)	32 (8.1%)	14 (4.7%)	137 (54.4%)	NA	NA
Reference	Van Rossum (2) yet unpublished	Gibson (3)	Rahman (4)	Gasparrini (5)	Taft (6)	Pärnänen (7)

^1^*n* (%); Median (IQR); DOL = Day of Life; *N* = number of samples or subjects, as indicated.

## Supplementary Material

Supplementary Material Figure S1

## Data Availability

All annotated data, metadata, and analysis scripts are available at https://github.com/RebeccaLuise/s_aureus DOI 10.5281/zenodo.17603738 with raw sequences available upon request.
