# Correction

**DOI:** 10.1080/19490976.2026.2620281

**Published:** 2026-01-23

**Article title**: *Staphylococcus aureus* colonization and bloodstream infection in very preterm infants

**Authors**: Knoll, R. L., Podlesny, D., Fortmann, I., Göpel, W., Zemlin, M., Lynch, S., Bork, P., Gehring S., & Härtel, C.

**Journal:** *Gut Microbes.*

**Bibliometrics:** Volume 17, Number 1, pages 1–7.

**DOI:** http://doi.org/10.1080/19490976.2025.2592423

In the published article, coding errors were identified affecting the assignment of samples to day-of-life categories in the meta-analysis. Specifically, for 3 out of the 6 included studies, the day-of-life was incorrectly computed, leading to misclassification of samples into incorrect day-of-life categories.

This error has been corrected, and all samples reclassified accordingly. As a result, 10 out of 1,782 samples were excluded from the revised analysis. Following these corrections, the meta-analysis was repeated.

The main conclusions of the study remain unchanged. However, the corrections affect the results displayed in **Figure 1d and 1e**, **Figure 2b, 2c, and 2d**, as well as the corresponding sections of **Table 2**, which have been updated accordingly.

