# Supplementary material for: Staphylococcus aureus colonization and bloodstream infection in very preterm infants
Source: Gut Microbes. 2025 Dec 2;17(1):2592423. doi: 10.1080/19490976.2025.2592423 (PMC12674446; doi:10.1080/19490976.2025.2592423)
Supplement: Supplementary Material — Figure S1 [file KGMI_A_2592423_SM1698.docx]

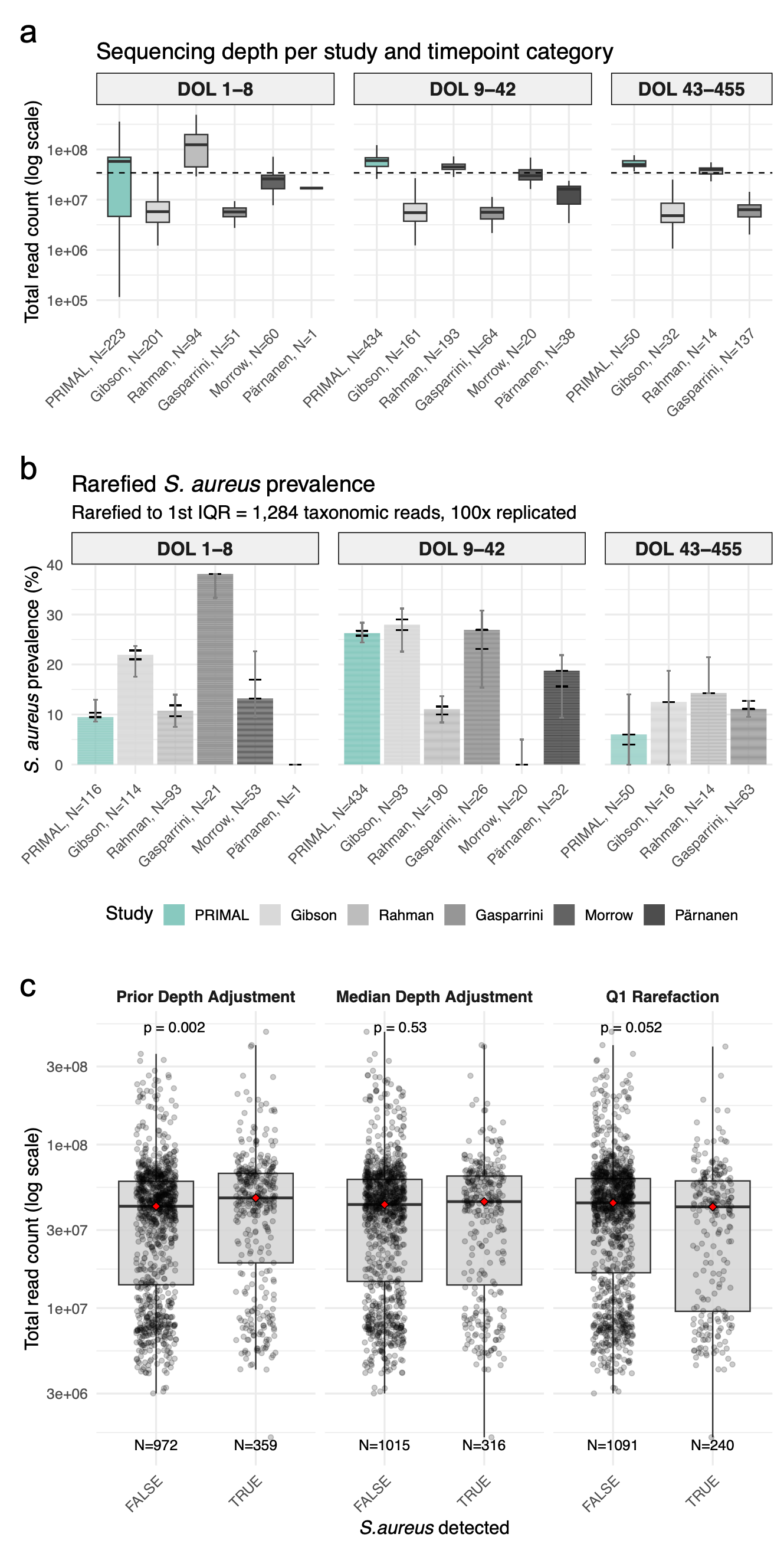


**Supplementary Figure 1: Sequencing depth heterogeneity and prevalence adjustment approaches.**
 **a.** Distribution of sequencing depth across studies and timepoints, highlighting substantial heterogeneity in sequencing effort. Because presence/absence (prevalence) estimates from metagenomic data are sensitive to stochasticity at low sequencing depth, direct comparisons between studies or timepoints without adjustment are biased. To address this, in the main manuscript we present a depth-adjusted approach, where taxon read counts are rescaled to a standardized sequencing depth prior to calling presence (≥1 read). This method minimizes stochastic dropout while preserving comparability across heterogeneous datasets.
 **b.** For comparison, prevalence of *S. aureus* was also calculated using a rarefaction-based approach, where all samples were subsampled to the first quartile (Q1) of sequencing depth across the dataset. This method accounts for differences in sequencing effort by equalizing read counts across samples, at the cost of discarding samples with lower sequencing depth than Q1. Despite this loss, prevalence estimates from rarefaction at Q1 sequencing depth strongly correlated (R = 0.95, Pearson correlation) with those obtained using the read count adjustment approach at the same depth (data not shown). This illustrates that both strategies effectively mitigate biases introduced by uneven sequencing depth, though the adjustment method presented in the main text avoids data loss inherent to rarefaction.

**c,** Relationship between *S. aureus* prevalence and sequencing depth. Boxplots show total read counts (log scale) stratified by *S. aureus* detection status across different methods. Points represent individual samples, and red diamonds indicate medians. Sample sizes (N) are shown below each category and are limited to samples that passed Q1 for rarefaction. Wilcoxon tests compare groups and resulting p-values are depicted. Rarefaction to the first quartile of sequencing depth (100 iterations) and depth-adjustment both remove dependence of prevalence estimates on sequencing depth, demonstrating the robustness of these approaches.
